# Internet use and livelihood risk perception: exploring the dynamic relationship and moderating factors between information dissemination and individual perception

**DOI:** 10.3389/fpsyg.2025.1479491

**Published:** 2025-09-23

**Authors:** Xiaoli Gan, Shuhan Yang, Yaqian Liu, Quan Yu

**Affiliations:** ^1^School of Business, Guilin University of Electronic Technology, Guilin, China; ^2^School of Mathematics and Statistics, Qiannan Normal University for Nationalities, Duyun, China

**Keywords:** livelihood risk perception, internet use, amplification effect, human capital, social capital, long-term impact

## Abstract

**Introduction:**

While the Internet brings development dividend to economy and society, it also changes the subjective attitude and risk perception of social members, which makes the complexity and uncertainty of economic society increasingly obvious. The development of economy and society is directly reflected in the livelihood issues, and consequently the widespread application of Internet has produced complex social effects on public perception of livelihood risks. This study explores the dynamic relationship and moderating factors between the Internet use and public perception of livelihood risks, considering various characteristics among different groups.

**Methods:**

This study employs the data of China Family Panel Studies (CFPS) from 2014 to 2020, conducted on respondents aged between 16 and 65, which captures the majority of Internet users in China. Specifically, the analysis aims to examine the direct impact of Internet use on public livelihood risk perception, alongside the moderating influence of human capital and social capital. Moreover, the research discerns variations in the impact across different groups.

**Results:**

Our findings reveal that Internet use significantly enhances the level of public livelihood risk perception, in which human capital and social capital exhibit a negative moderation effect. Furthermore, our findings highlight the heterogeneous effects of Internet use across diverse groups in livelihood risk perception. In addition, it is showed a steady decreasing tendency in the amplification effect of Internet on livelihood risk perception, while the COVID-19 made it temporarily rebound.

**Discussion:**

This study explores the relationship between the Internet and public livelihood risk perception, as well as its dynamic changes, and to analyze how to effectively manage and mitigate the risks arising in the digital age. This extension not only enriches the theory system about the social amplification of risk, but also provides theory guidance for effective risk management. What’s more, this study reveals the weakening amplification effect of Internet on livelihood risk perception and fills the gap in most literature that only focuses on the negative result of the absolute amplification effect of Internet on social risks.

## 1 Introduction

Risk has become the basic element of modern society and political agenda since the 1990s (Beck, 1992)^1^, and remains a priority for the present and future (Andreas and Ortwin, 2019). According to the Social Amplification of Risk Framework (SARF), risk is a social construct composed of information processes, social systems and individual responses (Kasperson et al., 1988). Worldwide, the Internet is continuously reshaping the ways of social interaction, resource flow, and information sharing, profoundly affecting the construction and dissemination of risks, which makes the complexity and uncertainty of economic society increasingly obvious. The development of economy and society is directly reflected in the livelihood issues concerning on pension, medical care, education, employment, environment, housing and so on. The popularity of Internet has made all kinds of livelihood services more convenient and efficient, and increased the public’s sense of livelihood gains. While the Internet brings development dividend to economy and society, it also changes the subjective attitude and risk perception of social members. The widespread application of Internet has produced complex social effects on public perception of livelihood risks. On the one hand, livelihood risks are the objective existence. The World Economic Forum’s Global Risks Report in 2024 leverages nearly two decades of date from the Global Risks Perception Survey (GRPS) and reveals a key trend that the main risks to the whole world in the medium and long term are increasingly concentrated in the field of livelihood. The spread of Internet has expanded the channels for the access and communication of risk information, with risks gradually being amplified in the process of dissemination through Internet media, thereby forming collective anxiety (Mark and Murphy, 2018). On the other hand, with the extensive application of information, network, digitalization and intelligence technologies, there are different types of digital divide among groups from varied socio-economic status, which exacerbates the degree of social inequality more likely to generate widespread livelihood anxiety (Scheerder et al., 2017; Frydman et al., 2021; Zheng and Walsham, 2021). The public’s differentiated risk perception will lead to different attitudes and behaviors, and even result in serious risk consequences (Pennings-Joost and Grossman, 2008). Therefore, with the coexistence of the Internet era and the risk society, it is necessary to deeply explore the relationship between the Internet and public livelihood risk perception, as well as its dynamic changes, and to analyze how to effectively manage and mitigate the risks arising in the digital age.

While economic development and technical advance have greatly raised the living standard of the public, the vulnerability of society has also grown with the soaring risks about environment, health and epidemic outbreak. The subjective construction of modern risk determines risk perception as the important research content in risk governance. Research on the influence factors of risk perception mainly starts from the psychometric paradigm, cultural paradigm as well as social paradigm. The first is the psychometric paradigm proposed by Slovic (1987), which emphasizes that risk is the individual subjective recognition influenced by multiple aspects in psychology, society, institution and culture. Hakes and Viscusi (2004) argued that individuals with adequate knowledge had more accurate risk beliefs, and there are significant differences in the risk perception by race and gender. The second is the cultural paradigm represented by the cultural theory of risk centered on Douglas, which believes that risk is ultimately the phenomenon of social construct and varies depending on the outlook people insist, with trust being an important component. Kasperson (2012) points out to trust one of the pathways affecting risk perception, and a lack of public trust in relevant institutions and managers can amplify their perception of risk. The third is the social paradigm which holds that social factors are posited to exert a more profound influence on risk perception than physiological and psychological factors, primarily involving the role of media, the viewpoints of experts and policy institutions (Rogers, 1997; Wachinger et al., 2013). As the research goes further, the stated three paradigms tend toward to converge, forming two representative theories: the cultural cognition of risk and the social amplification of risk. The social amplification of risk happens in two phases: the transmission of risk information and the response of social institution, that is, risk information begins with dissemination through social amplification sites such as government, media, and the Internet, and then transmitted by individual amplification stations like cognition, attitude and trust, thereby affecting a wider range of groups.

Nowadays, the Internet is generally considered as an important social site to amplify risks through interactions among various sites (Chung, 2011). Under such background, several scholars have proceeded the relationship between the Internet and public risk perception, and further discussed the role that trust plays in this context (Hong and Zhang, 2020; Bland et al., 2024; Liu et al., 2024). At present, there are quite a number of problems to be further investigated and explored in the impact of Internet use on public risk perception: (1) Previous studies may be short-term owing to one round of survey data, lacking consideration for long-term impacts (Moon and Shim, 2019). (2) Previous studies are mostly concerned with the impact of Internet use on a specific type of risk perception, neglecting the social risks arising from the accumulation of modern risks that emerge as co-product of economic and social development. There is little attention paid to the relationship between Internet use and livelihood risk perception (Hilverda et al., 2017; Liu et al., 2021). (3) Previous studies have discussed mediation effect within the amplification framework of media, but the potential specific changes and conditional relationships that may exist behind amplification phenomenon have not been excavated (Liu et al., 2024; Xu et al., 2024). (4) Previous studies focus on the absolute amplification effect of Internet on risk perception without in-depth discussion on whether the amplification effect is enhanced or weakened (Jing, 2023).

As China is a country with the largest number of Internet users in the world, its economic and social development must be deeply affected by the Internet. Meanwhile, the contradiction about imbalanced and insufficient development of livelihood construction in China is still outstanding, creating complex social risks. Consequently, taking China as a case study to explore the dynamic relationship and moderating factors between the Internet and public livelihood risk perception can provide valuable insights and strategies for global risk management, especially for developing countries with a vast rising space in the development of Internet, and such countries need to address similar risk challenges in terms of Internet penetration and construction. The findings of this study can draw on the reference for risk management in these countries. We utilize four-period data of the China Family Panel Studies (CFPS) from 2014 to 2020 and construct a comprehensive indicator of livelihood risk perception from eight dimensions including government integrity, social security, employment, education, medical care, housing, environment protection, and wealth inequality. Our findings reveal that the amplification effect of Internet on livelihood risk perception significantly exists with robustness after addressing endogeneity and replacing the indicator construction method, and human capital and social capital can mitigate this amplification effect. The effect and mechanism remain consistent in the long term. Specifically, the impact of Internet use on the public livelihood risk perception varies significantly among different groups, more obvious in those without university degree, with middle income, in the middle and elder age as well as the urban area. In addition, the amplification effect is on track to decline steadily, while the COVID-19 made it temporarily rebound. But it has not significantly deviated from the appropriate range in the long term.

Therefore, this study makes the following marginal contributions: (1) In the research content, this study explores the moderation effect of human capital and social capital on the relationship between Internet use behavior and individual livelihood risk perception. This extension not only enriches the theory system about the social amplification of risk, but also provides theory guidance for effective risk management. (2) As far as the research perspective is concerned, this study reveals the dynamic change process of the amplification effect of Internet on public livelihood risk perception from a long-term perspective. It is found that the amplification effect shows a decreasing trend. And this study focuses on the gradually weakening amplification effect, instead of the negative influence of the absolute amplification effect of Internet on social risks.

The remainder of this article is structured as follows: Section 2 introduces the amplification effect of Internet on livelihood risk perception and puts forward relevant research hypotheses. Section 3 describes the data sources and variable settings employed in the study. Section 4 presents an empirical investigation of the long-term effects and specific mechanisms about Internet on the livelihood risk perception. Finally, Section 5 provides analysis and conclusions, as well as long-term policy recommendations for risk management based on empirical analysis.

## 2 Theoretical analysis and research hypotheses

### 2.1 The amplification effect of internet on livelihood risk perception

Risk perception has been a core issue in the field of risk analysis and management, referring to the subjective feelings about risk evaluated by individuals in the conditions of insufficient information and uncertainty (Slovic, 1987; Sitkin and Pablo, 1992). By extension, livelihood risk perception pertains to the subjective assessment of public made toward livelihood risks with the basis of self-defined standards, which can be explained from the aspect of the “absolute gains” and “relative gains” obtained by the public. On the one hand, the absolute gains serve as a foundation for mitigating livelihood risk perception. If people have not received tangible benefits from economic development, their perception of risks related to livelihood may intensify. On the other hand, social members mostly base their subjective evaluations on the “relative gains” perceived through social comparison, encompassing self-time comparison between past and present as well as horizontal comparison among groups (Albert, 1977). The outcomes of subjective cognition in social comparison are closely tied to public risk perception, and individuals’ subjective perception of livelihood risks may be enhanced with the gradually widening gap in livelihood security between themselves and others. Studies have indicated that the influence and effect of information in modern society become clearer as the popularization of information technology and the Internet, then risk information spreading rapidly on the Internet will affect individuals’ judgment of the resources they currently hold (Oh et al., 2014; Seabrook et al., 2016; Castellacci and Tveito, 2018). Therefore, the extent to which the widespread use of Internet influences the formation and evolution of public livelihood risk perception is worth to explore in depth. At present, the Internet is changing and reshaping the social form in an all-round way, which exerts a profound impact on all aspects of society development. According to the theory of social acceleration raised by Hartmut Rosa, the emergence of new technologies is fundamentally altering former lifestyle and previous forms of social interaction, hastens the steps of social changes as well as pace in people life. Such demand for the fast-paced life continuously propels the accelerated development of new techniques in turn, thus forming a closed and self-driven social circular system, among which the acceleration of communication, transportation, and production process constitutes three major driving mechanisms of society acceleration. This theory also holds strong explanatory power for the social changes and influences in China during the digital age. In the Chinese context of the society acceleration led by technological advancement, people are frightened about difficulties in keeping up with the pace of technological progress and social change. Moreover, the growth of objective benefit may not match the speed of social cycle, which can easily trigger individual anxiety. At the same time, there exists digital divide between different groups due to differences in the ability to grasp., control, and utilize information, and individual subjective risk perception may exhibit alienation as various accompanying forms of social pressure gradually emerge.

The phenomenon of information overload is an important reason for the alienation in the digital age. The information overload caused by the function of Internet to spread information rapidly and conveniently plays an important role in the process of the increasing perception of livelihood risks. The Internet has made a vast amount of negative information widely disseminated and spread, such as some ostentatious behaviors of social comparison, which may exacerbate the psychological gap after vertical self comparison and horizontal social comparison, leading people to perceive the gap in life quality to be larger than the actual difference. As a propagate media, network media plays an increasingly prominent role in this process. Compared with the traditional public media, Internet communication is characterized by mass information, decentralization, and cross-region dissemination. Coupled with the loose regulatory policy of government, the spread of risk information on the Internet has become even more rapid. The theory about risk communication holds that risk disseminators provide risk information with certain tendency, which promotes changes in individual risk attitude and perception (Rickard, 2019). The social amplification of risk formed by the rapid spread of livelihood risk information on the Internet, is likely to result in the deviation of self-cognition, thus changing the risk perception in social groups and generating widespread social anxiety. On the basis of the theoretical analysis above, we come forward with hypothesis 1:

*H1:* Internet use can strengthen public livelihood risk perception.

However, it is important to note that there have been significant improvements in public digital literacy and skills in recent years. These improvements are not only reflected in the identification of information but also in the rational assessment and response to risk information. For instance, when facing the grave public health event like COVID-19, as the government took effective prevention measures and enhanced the level of information disclosure, along with the growing adaptability of the public and the increasingly rational demand structure of information from Internet, the amplification effect of Internet on public risk perception quickly rebounded and had been falling steadily (Li et al., 2022). It can be inferred from this that public adaptability to risks and their digital literacy have been enhanced over time. Furthermore, the integrated effect of the continuous optimization of risk communication strategies, the strengthening of network supervision, the improvement of social trust as well as the rational attention of the public to risk information makes this “external boost” of the Internet on track to decline steadily. Thereby, hypothesis 2 is proposed:

*H2:* The amplification effect of Internet on livelihood risk perception shows a decreasing tendency.

### 2.2 The impact mechanism of internet use on livelihood risk perception

According to the characteristics of information interaction, the function of Internet can generally be categorized into two major types: human-machine interaction for information acquisition and human-machine-human interaction for social communication. The impact of information acquisition on public livelihood risk perception is influenced by individual learning ability and self-regulation capability, which means that human capital plays a significant role in this process. And the function of social communication relates directly to social capital such as social network, communal participation, trust, and reciprocity. By offering a variety of experience viewpoints and diverse sources of information, it assists individuals in building a more comprehensive and balanced framework of risk cognition, thus influencing public livelihood risk perception. Consequently, human capital and social capital take vital roles in the process by which Internet use affects public livelihood risk perception.

#### 2.2.1 Human capital

According to the theory of the second-level digital divide, different groups have different purposes to use Internet and capabilities to operate digital technology. In the process of Internet amplifying public livelihood risk perception, the level of human capital not only directly affects the possibility and frequency of Internet use but also significantly determines the proficiency in digital skills, ultimately influencing the extent of this amplification effect. First, Individuals with a higher level of human capital typically possess a higher socio-economic status (Currie, 2009), enjoying more comprehensive and faster network with greater acceptability for Internet. Second, thanks to a higher level of education, they can better adapt to the Internet development and make better use of the information on the Internet (Bonfadelli, 2002). These make them more likely to overcome the gaps in access and usage of Internet, so as to effectively identify, assess, and process risk information on the Internet (Hu and Xu, 2024). What’s more, a higher level of human capital makes individuals more conscious of using the Internet to promote their own development and obtain more effective information with the basic cultural literacy and operational skills, so as to better digest the impact brought by livelihood risks. In view of the above, hypothesis 3 is brought up:

*H3:* An increase in the level of human capital helps to mitigate the amplification effect of Internet on livelihood risk perception.

#### 2.2.2 Social capital

Bourdieu suggests that social capital encompasses the actual and potential resources associated with a network of enduring relationships that are mutually acknowledged. According to the formation mechanism of social capital, there are two forms of social capital, which are structural and cognitive (Bastelaer, 2000; Grootaert et al., 2004; Chou, 2005). The former is mainly manifested through participation in formal organizations, while the latter is primarily reflected in attitudes such as trust and reciprocity. Both enhance individual sense of social identity and facilitate the sharing of risk information as well as the coordination of risk response strategies (Kramer, 2006; Li et al., 2024). In reality, those with abundant social capital often have relatively steady relationship networks that are the important channels for public information dissemination (Uzzi, 1996; Hansen, 1999). This may reduce their dependence on the Internet as the main source to obtain information and support, thereby partly diminishing the impact of Internet on risk perception. On the contrary, individuals with lower level of social capital may rely more on the Internet to meet their needs for social participation and emotional support, which makes them more vulnerable to the amplification of risk on the Internet (Qian et al., 2022). In addition, social attitudes such as trust and reciprocity promote the sharing of risk information and the coordination of risk response strategies, and then reduce individual fear of risk and concern about uncertainty, further weakening the amplification effect of Internet on public livelihood risk perception. Thus, it can be seen that social capital squeezes out the use of Internet, and then plays a mitigating role in the process by which Internet amplifies public livelihood risk perception, that is, hypothesis 4:

*H4:* An increase in the level of social capital helps to weaken the amplification effect of Internet on livelihood risk perception.

Human capital and social capital exert the moderation effect on the relationship between Internet use and public livelihood risk perception. Higher the level of human capital and social capital, less the amplification effect of Internet on livelihood risk perception. Given that the accumulation of human capital and social capital has significant long-term effects on economic and social development, we propose hypothesis 5:

*H5:* Human capital and social capital exhibit a long-term negative moderation effect in the process of public livelihood risk perception enhanced by Internet use.

## 3 Methodology

### 3.1 Sample

The data utilized in this study is derived from the China Family Panel Studies (CFPS) that reflects the economy, society, and population development in China with the sample covering 95% of the total population. CFPS stands as one of the most authoritative and representative sources of microdata currently available in China.

In terms of sample selection, considering the focus of this study on the impact of Internet use on public livelihood risk perception, the analysis is conducted on respondents aged between 16 and 65 to ensure the validity of the data, which captures the majority of Internet users in China. In terms of time dimension, this study uses CFPS 2014, 2016, 2018, and 2020 survey data to test the above research hypotheses. The reason is that the time is approximately synchronous with the rapid spread of the Internet, which provides good data support for us to better test the change of public livelihood risk perception in the context of Internet popularization.

### 3.2 Measures

#### 3.2.1 Dependent variable

Public subjective feelings and cognitions regarding various risks in the livelihood domain constitute the main content of public livelihood risk perception. Livelihood issues mainly include three levels: the first level is survival status, involving compulsory education, public health, basic social security and housing security; The second level is livelihood sources, including employment equity and income distribution; The third level is the quality of life, which involves environmental protection and higher-level social welfare. Government integrity is an important guarantee for maintaining the basic living conditions, promoting fair employment, and improving the quality of life. It is both a cause of livelihood issues and a result of livelihood risks. Based on the research hypotheses and combined with the questionnaire of CFPS, this study measures public livelihood risk perception using the survey item “How serious do you think the following issues are in China” that covers eight aspects including government integrity, social security, employment, education, medical care, housing, environment protection, and wealth inequality, with options set on the scale from 0 to 10, where a higher score indicates that the respondent subjectively considers the corresponding livelihood issue to be more severe. To facilitate empirical analysis of the transformation in public livelihood risk perception, this study follows the approach of Xu and Lai (2021), first applies min-max normalization to standardize the eight indicators mentioned above, and then calculates the Cronbach’s Alpha value which is 0.872, indicating a high level of consistency. Finally, the standardized variables of the eight indicators are summed up to construct a comprehensive variable for public livelihood risk perception.

#### 3.2.2 Independent variable

This study defines the key independent variable as Internet use, measured by the binary indicator “whether the respondent uses the Internet or not.” The statistical results show that the Internet usage in 2020 was 73% consistent with the annual Internet penetration published by the China Internet Network Information Center (CNNIC)^2^, indicating that the data is of great representability and reliability.

#### 3.2.3 Moderating variables

The moderating variables in this study include human capital and social capital. Human capital refers to the total stock of knowledge, skill, ability, and health quality that present in humans with economic value. Existing research generally approaches the study of human capital from education and health, but neglects the skill aspect. For this reason, this study incorporates the skill component of human capital into the model, along with education and health, to establish a measurement system for human capital, in which the indicators contain education years, health status, and work experience used as a proxy variable for skill. Since the object of this study is micro-individual with high discreteness, we follow the method of Tian (2023) and uses the entropy method to measure human capital so that its level of each individual can be more accurately and objectively quantified. In the research of social capital, due to the fact that Chinese concept of relationships is not equivalent to the connotation of Western social capital, there may be a problem of conceptual mismatch in measurement such as family relationships, gifts and favors. As a result, this study defines social capital as the value of networks, norms, and social organizations through which individuals coordinate and cooperate externally. Taking into account of the reality in China, we use the respondent’s membership in organizations to represent social capital, as joining different organizations can reflect the social and economic resources that can be utilized and mobilized to a certain degree. Membership in organizations is generally considered one of the most important and reliable indicators of social capital because it reflects the individual interest in public affairs and inclination to contribute to the public welfare (Guiso et al., 2016).

#### 3.2.4 Control variables

Control variables consist of the individual and provincial levels. Based on existing research (Pu and Zhao, 2021), the public livelihood risk perception is affected by individual factors such as age, gender, education, marital status, health status, region, participation in medical insurance and endowment insurance, income, economic and social status, life satisfaction, confidence about the future, and household scale. Therefore, these main variables are selected for control in the regression. Besides, since the public livelihood risk perception index encompasses factors such as education, social security, employment, medical care, environment protection and housing, we match the corresponding expenditure of public livelihood in the province where the respondent is located and control for its logarithm. The definitions of all variables are shown in Table 1.

**Table 1:** Definitions of variables.

	Variables	Definition
Dependent variable	Risk_perception	The respondent’s perception of the extent of livelihood issues
Independent variable	Internet	1 for using, 0 for no using
Moderating variables	Human_capital	A comprehensive variable composed of education years, health status, and work experience
	Social_capital	Whether the respondent is an organizational member.
Control variables at the individual level	Age	Age of the respondent
	Gender	Gender of the respondent; 1 for male, 0 for female
	Marriage	Whether the respondent is married;1 for yes, 0 for no.
	Education	Respondent’s education years
	Scale	The scale of the respondent’s household
	Work	Whether the respondent is employed;1 for yes, 0 for no.
	Urban	1 for urban areas, 0 for rural areas
	Health	1 for very unhealthy, 5 for quite healthy
	Medical_insurance	Whether respondent holds medical insurance; 1 for yes, 0 for no.
	Endowment_insurance	Whether respondent holds endowment insurance; 1 for yes, 0 for no.
	Income	Natural log of the total annual respondent’s income
	Economic status	1 for very low, 5 for quite high
	Social status	1 for very low, 5 for quite high
	Life satisfaction	1 for very dissatisfied, 5 for quite satisfied
	Confidence level about the future	1 for very discouraged, 5 for quite confident
Control variables at the provincial level	Education expenditure	Natural log of education expenditure in the province where the respondent is located
	Social security and employment expenditure	Natural log of social security and employment expenditure in the province where the respondent is located
	Medical and hygienic expenditure	Natural log of medical and hygienic expenditure in the province where the respondent is located
	Environmental expenditure	Natural log of environmental expenditure in the province where the respondent is located
	Housing security expenditure	Natural log of housing security expenditure in the province where the respondent is located

## 4 Empirical analysis

### 4.1 Descriptive statistical analysis

Table 2 introduces the statistical analysis of the full sample, and lists the descriptive characteristics for each variable stratified by Internet use. The average of livelihood risk perception for the full sample reaches 5.026, indicating that the livelihood risk perception of most respondents is above mean level. There are certain differences in livelihood risk perception and control variables between the two groups. Notably, the average risk perception of Internet users is significantly higher than that of non-users, which provides a basic understanding and intuitive judgment for the following empirical tests. The average age of the respondents is approximately 41 years old, with Internet users being markedly younger than non-users. Moreover, the majority of Internet users live in urban areas with higher levels of education and income. In the aspect of capital accumulation, compared to non-users, Internet users have a higher level of social capital, while the level of human capital is lower, suggesting that the Internet should keep developing with the improvement of users’ human capital in step and it is important to reduce disparities in social capital during the process of Internet popularization.

**Table 2:** Descriptive analysis.

Variables	Full sample				Users	Non-users	Diff.
	Min	Max	Mean	Std. Dev.	Mean	Mean	
Risk_perception	0.000	8.000	5.026	1.563	5.202	4.547	0.655^***^
Internet	0	1	0.730	0.443	–	–	
Human_capital	0	1.000	0.524	0.172	0.496	0.600	−0.104^***^
Social_capital	0	1	0.300	0.456	0.342	0.167	0.175^***^
Age	16	65	41.060	13.663	37.018	52.040	−15.022^***^
Gender	0	1	0.500	0.500	0.514	0.464	0.050^***^
Marriage	0	1	0.760	0.427	0.693	0.941	−0.248^***^
Education	0	22	9.290	4.406	10.560	5.837	4.723^***^
Scale	1	15	4.300	2.022	4.244	4.443	−0.199^***^
Work	0	1	0.790	0.409	0.806	0.738	0.068^***^
Urban	0	1	0.500	0.500	0.550	0.373	0.177^***^
Health	1	5	3.190	1.163	3.290	2.910	0.380^***^
Medical_insurance	0	1	0.900	0.304	0.890	0.914	−0.024^***^
Endowment_insurance	0	1	0.680	0.468	0.654	0.737	−0.083^***^
Income	0.000	15.610	11.224	1.034	11.361	10.850	0.511^***^
Economic status	1	5	2.750	1.089	2.650	3.010	−0.360^***^
Social status	1	5	2.980	1.020	2.867	3.291	−0.424^***^
Life satisfaction	1	5	3.950	0.926	3.900	4.100	−0.200^***^
Confidence level about the future	1	5	4.140	0.911	4.118	4.185	−0.067^***^
Education expenditure	5.340	8.160	7.116	0.549	7.111	7.127	−0.016^***^
Social security and employment expenditure	5.220	7.600	7.049	0.416	7.047	7.053	−0.006^***^
Medical and hygienic expenditure	4.780	7.480	6.518	0.513	6.512	6.536	−0.024^***^
Environmental expenditure	3.910	6.250	5.362	0.535	5.362	5.364	−0.002^**^
Housing security expenditure	3.730	6.500	5.450	0.464	5.448	5.455	−0.007^***^
*N*	19,084				13,954	5,130	

*p < 0.1; **p < 0.05; ***p < 0.01.

### 4.2 Benchmark regression analysis

The regression outcome of Internet use on public livelihood risk perception (see Table 3) presents that Internet use significantly strengthens public livelihood risk perception at 1% level, which confirms H1. Concretely, if an individual uses the Internet, the level of livelihood risk perception significantly increases by 0.424. Given that the range of values for the livelihood risk perception variable obtained by summarizing after standardization is [0,8], this influential effect is statistically significant. The economic meanings of other coefficients are similar. In conclusion, the Internet has an amplification effect on the public livelihood risk perception in the modern society with great acceleration.

**Table 3:** Baseline regression results.

Variables	Risk_perception
	(1)
Internet	0.424^***^
	(0.031)
Age	−0.005^***^
	(0.001)
Gender	−0.039^*^
	(0.023)
Marriage	0.050^***^
	(0.019)
Education	0.030^***^
	(0.003)
Scale	0.019^***^
	(0.006)
Work	−0.059^*^
	(0.032)
Urban	0.008
	(0.025)
Health	−0.037^***^
	(0.011)
Medical_insurance	0.027
	(0.038)
Endowment_insurance	0.035
	(0.027)
Income	0.014
	(0.012)
Economic status	−0.005^***^
	(0.001)
Social status	−0.061^***^
	(0.013)
Life satisfaction	−0.066^***^
	(0.015)
Confidence level about the future	0.086^***^
	(0.015)
Education expenditure	0.164
	(0.103)
Social security and employment expenditure	0.382^***^
	(0.047)
Medical and hygienic expenditure	−0.549^***^
	(0.121)
Environmental expenditure	0.159^***^
	(0.040)
Housing security expenditure	−0.120^**^
	(0.053)
Constant	3.900^***^
	(0.247)
Obs	19,084
Adj. R^2^	0.052
VIF	4.39

***,**,* indicate statistically significant at the level of 1%, 5% and 10% respectively, the same below..

### 4.3 Endogeneity test

#### 4.3.1 Instrumental variable estimation

This study attempts to test the impact of Internet use on public livelihood risk perception, but there may be endogenous problems. Firstly, the two-way causality between Internet use and public livelihood risk perception leads to endogenous problems. On the one hand, the Internet will amplify the discussion on livelihood issues, thus strengthening individual risk perception; On the other hand, people who are worried about livelihood issues may be more likely to use the Internet to search for relevant information. Secondly, the correlation between disturbances and explanatory variables can arise from omitted variables, which can result in endogeneity issues. To address this problem, the instrumental variable is designed as the Internet usage average of others in the same community or village, that is Internet_mean, with application of two-stage least square (2SLS) method. On the one hand, the variable represents the degree of Internet penetration related to the Internet use behavior of respondents in this area. On the other hand, the primary reason why Internet can strengthen public livelihood risk perception is that the use of Internet, which means that it is difficult for the Internet penetration to affect the public livelihood risk perception through approaches other than Internet use. This variable conforms to the requirements about correlation and exogeneity of instrumental variable as its validity confirmed by statistical tests (see Table 4), and the regression results of the instrumental variable are shown in Table 5, consistent with the results before using the instrumental variable method. Moreover, the regression results in Table 5 show that the estimated coefficient for Internet use is 1.74 (*p* < 0.001), significantly higher than 0.424 (p < 0.001) in Table 3. This indicates that the parameter estimation of the ordinary least squares (OLS) method is biased, and the amplification effect of Internet on livelihood risk perception would be severely underestimated without accounting for endogeneity. Therefore, the 2SLS analysis once again verifies the robustness of Internet in strengthening public livelihood risk perception.

**Table 4:** Instrumental variable test.

Variables	Risk_perception
Instrumental variable	Internet_mean
Suspected endogenous variable	Internet
Weak identification test:	
Cragg–Donald Wald Fstatistic	387.616
Stock–Yogo weak ID test critical value	16.380 (10%)
Tests of endogeneity:	
Wu–Hausman F	63.439
Chi-sq *p*-value	0.000

**Table 5:** Endogeneity test results.

Variables	Risk_perception
	(1)
Internet	1.740^***^
	(0.226)
Age	0.011^***^
	(0.004)
Gender	−0.110^***^
	(0.026)
Marriage	0.009^*^
	(0.005)
Education	−0.010
	(0.012)
Scale	0.025^***^
	(0.007)
Work	−0.022^***^
	(0.008)
Urban	0.009
	(0.007)
Health	−0.041^***^
	(0.013)
Medical_insurance	0.041
	(0.040)
Endowment_insurance	−0.046
	(0.031)
Income	−0.047^***^
	(0.018)
Economic status	−0.002^*^
	(0.001)
Social status	−0.035^**^
	(0.016)
Life satisfaction	−0.056^***^
	(0.017)
Confidence level about the future	0.083^***^
	(0.016)
Education expenditure	0.087
	(0.116)
Social security and employment expenditure	0.342^***^
	(0.054)
Medical and hygienic expenditure	−0.400^***^
	(0.141)
Environmental expenditure	0.154^***^
	(0.045)
Housing security expenditure	−0.106^*^
	(0.058)
Constant	3.041^***^
	(0.332)
Obs	19,084

***, **, *indicate statistically significant at the level of 1, 5 and 10% respectively.

#### 4.3.2 Difference-in-difference estimation

To further exclude the influence of unobservable factors on the relationship between Internet use and public livelihood risk perception, this study employs the following experimental design to preliminarily test whether the amplification effect of Internet on public livelihood risk perception is an independent impact. Owing to the unprecedented influence of COVID-19 in 2020, the public livelihood risk perception at multiple levels has been affected by health risk, economic risk, the tension supply of specific resources and so on, potentially masking the independent impact of Internet use on public livelihood risk perception. Besides, significant changes in the frequency and purpose of Internet use may have occurred during the pandemic, which makes the amplification effect of Internet on livelihood risk perception mixed with behavior changes related to the pandemic rather than an independent impact. Therefore, this study takes 2014 and 2018 as the sample examination periods and sets the control group as those not using the Internet in both years, and the experimental groups include those from non-users to uses as well as those using the Internet in both years. By comparing the differences in livelihood risk perception between the two experimental groups and the control group, the role of Internet can be separated from the impact of other factors to a certain extent. In detail, based on the data of CFPS in 2018, the independent variable “Internet use_1” is reconstructed, with 0 for respondents who did not use the Internet in both 2014 and 2018, and 1 for those who used it in both years; “Internet use_2” is also defined as 0 if respondents did not use the Internet in both years, otherwise is 1 if they shifted from non-users to uses. By controlling other conditions to be consistent, regression analysis is conducted on the dependent variable and the newly constructed independent variables (see Table 6).

**Table 6:** Regression results excluding the influence of other factors.

Variables	Risk_perception	Risk_perception	Risk_perception
	(1)	(2)	(3)
Internet_1	0.497^***^		
	(0.038)		
Internet_2		0.410^***^	
		(0.032)	
Internet_3			0.067^**^
			(0.026)
Age	−0.015^***^	−0.013^***^	−0.010^***^
	(0.002)	(0.001)	(0.001)
Gender	0.026	0.031	0.016
	(0.028)	(0.026)	(0.025)
Marriage	0.048^***^	0.043^***^	0.044^***^
	(0.012)	(0.008)	(0.007)
Education	0.016^***^	0.018^***^	0.069^***^
	(0.006)	(0.006)	(0.009)
Scale	−0.005	0.001	−0.007
	(0.007)	(0.006)	(0.006)
Work	−0.002	−0.008	−0.002
	(0.007)	(0.006)	(0.005)
Urban	0.004	0.005	0.010
	(0.013)	(0.010)	(0.009)
Health	−0.019	−0.013	−0.042^***^
	(0.012)	(0.011)	(0.013)
Medical_insurance	0.027	0.048	0.059
	(0.055)	(0.049)	(0.046)
Endowment_insurance	0.074^**^	0.020	−0.011
	(0.034)	(0.031)	(0.030)
Income	0.041^***^	0.043^***^	0.050^***^
	(0.014)	(0.014)	(0.015)
Economic status	−0.002^*^	−0.001	−0.002^**^
	(0.001)	(0.001)	(0.001)
Social status	−0.024^**^	−0.044^***^	−0.050^***^
	(0.010)	(0.009)	(0.009)
Life satisfaction	−0.085^***^	−0.054^***^	−0.054^***^
	(0.017)	(0.016)	(0.016)
Confidence level about the future	0.076^***^	0.057^***^	0.038^**^
	(0.017)	(0.016)	(0.017)
Education expenditure	0.496^***^	0.504^***^	0.056
	(0.166)	(0.153)	(0.147)
Social security and employment expenditure	0.440^***^	0.418^***^	0.391^***^
	(0.053)	(0.048)	(0.049)
Medical and hygienic expenditure	−1.165^***^	−1.185^***^	−0.600^***^
	(0.183)	(0.168)	(0.162)
Environmental expenditure	0.388^***^	0.442^***^	0.358^***^
	(0.051)	(0.047)	(0.046)
Housing security expenditure	−0.185^***^	−0.162^***^	−0.210^***^
	(0.062)	(0.056)	(0.054)
Constant	4.933^***^	4.661^***^	5.072^***^
	(0.327)	(0.296)	(0.294)
Obs	11,169	13,321	11,858
Adj. R^2^	0.090	0.066	0.035

***, **, *indicate statistically significant at the level of 1, 5 and 10% respectively.

It can be found that the livelihood risk perception of respondents with continued use of the Internet, as well as those who have experienced the transition from non-users to uses, is higher than that of those never using the Internet. Subsequently, we characterize those with the transition from non-users to uses as the control group assigned a value of 0, and those who used the Internet in both years as the experimental group assigned a value of 1, constructing a new independent variable “Internet use_3.” The results show that the livelihood risk perception of the experimental group is still higher than that of the control group, which further supports H1.

**Figure 1:**
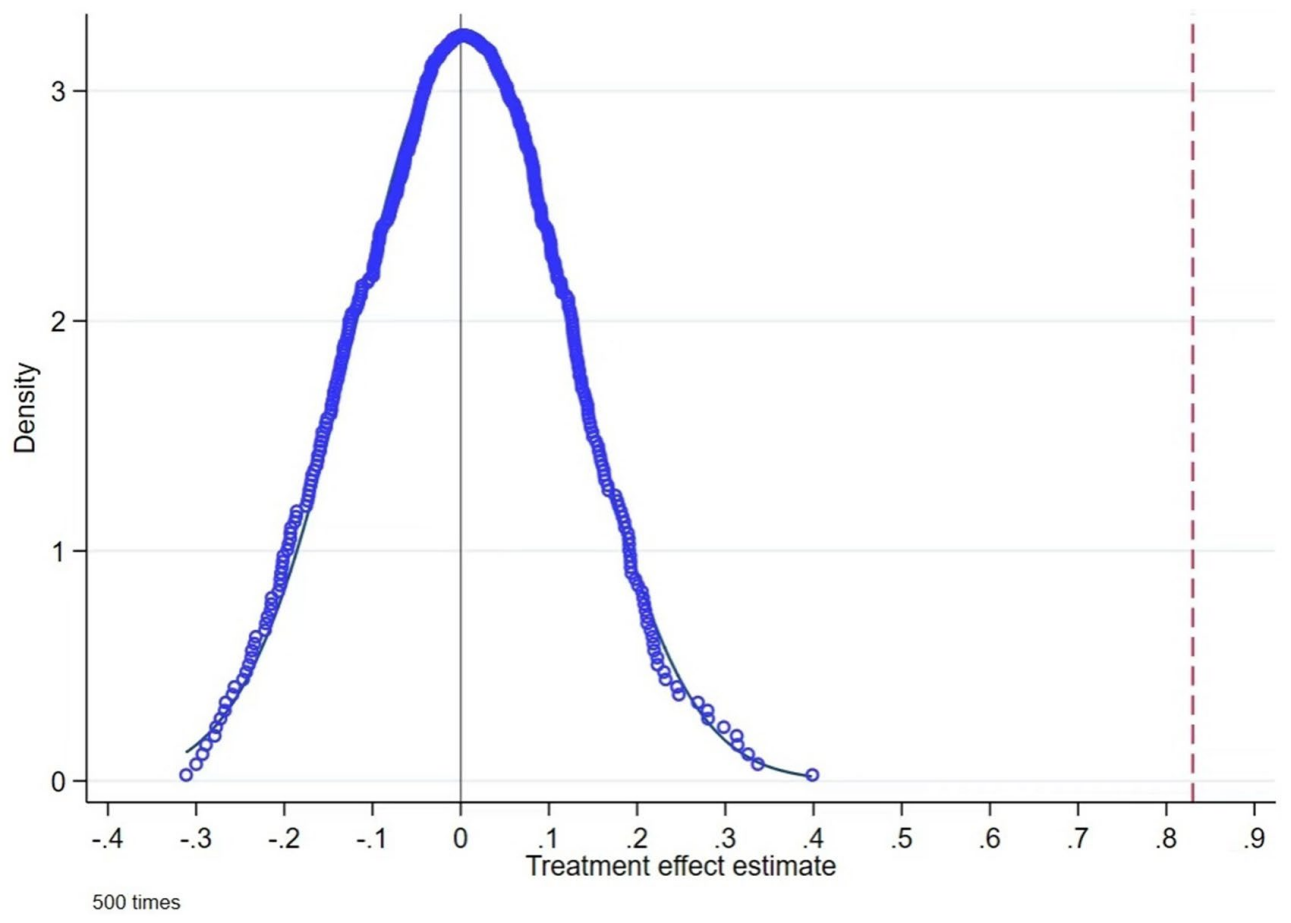
Kernel density estimation of placebo test.

Due to limitations in the temporal span of the experimental data and challenges in controlling for heterogeneous household characteristics, this experimental design cannot completely eliminate the estimation bias caused by unobserved factors. To address this concern, we conduct a more rigorous placebo test on the “Internet use” variable. Specifically, unlike the benchmark regression that defines the treatment and control groups based on the criterion of “whether to use the Internet,” this test randomly assigns half of the baseline sample to a pseudo-treatment group and the remaining half to a pseudo-control group, constructing a “placebo treatment” dummy variable for regression analysis. The purpose is to examine whether the amplification effect of Internet on livelihood risk perception stems from unobserved confounding factors captured by the placebo variable. We repeat this random assignment process 500 times and perform kernel density estimation on the placebo coefficients. As shown in Figure 1, the placebo coefficients from random assignments approximately follow a normal distribution centered around zero, while the baseline estimate lies far outside this distribution. This demonstrates that the risk-amplifying effect identified in the baseline regression is indeed attributable to Internet use rather than unobserved confounding factors or random factors, supporting the robustness of our empirical findings.

### 4.4 Heterogeneity

**Figure 2:**
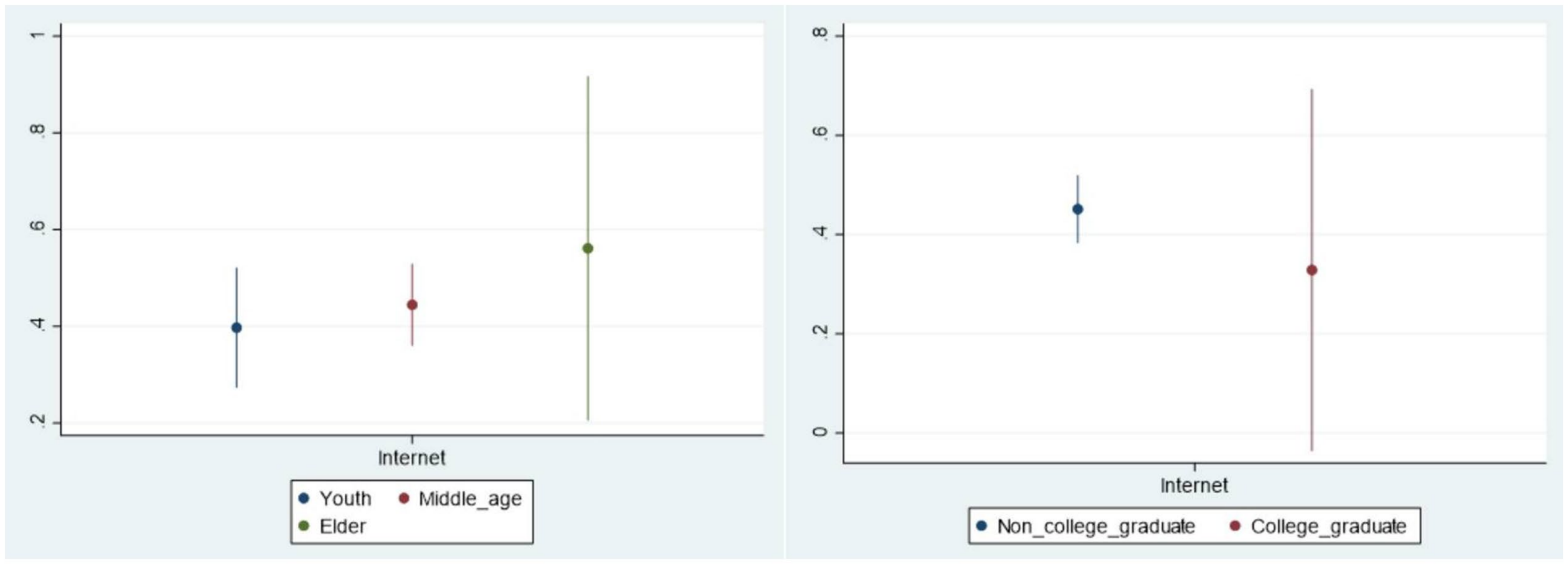
Heterogeneity of age and education level.

**Figure 3:**
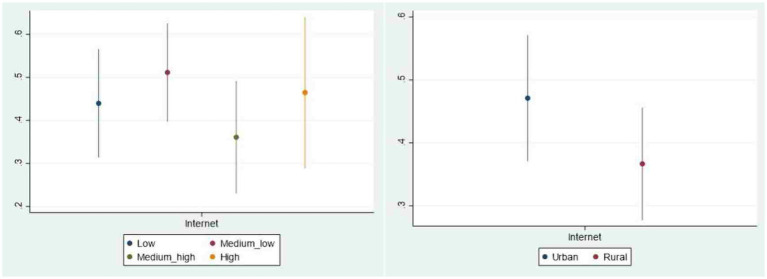
Heterogeneity of urban-rural and income.

The benchmark regression analysis about the relationship between Internet use and public livelihood risk perception is based on the average level. In order to better understand the differences related to the characteristics of various groups, we examine the heterogeneity of the impact of Internet use on the livelihood risk perception in different age groups, different education levels, different income levels as well as different regions. This study displays the analysis of heterogeneity with graphical representations for more intuitive observation (See Figures 2, 3). The vertical axis represents the average marginal effect of Internet use on public livelihood risk perception, and the horizontal axis is the Internet use variable in different samples. On the whole, the impact of Internet use on public livelihood risk perception varies among different groups.

#### 4.4.1 The heterogeneity of age

The heterogeneity of age indicates that the amplification effect of Internet on public livelihood risk perception is most evident in the elderly, followed by the middle-aged population, and the last is the young. Possible reasons can be explained as follows: On the one hand, due to a lower Internet penetration among the elderly compared with middle-aged and young people in the sample data, the marginal effect of Internet use is higher in the elderly. On the other hand, China has gradually entered into the aging society, and the elderly have more urgent demands for livelihood security, such as issues related to pension security and medical care making their livelihood risk perception more susceptible to the impact of Internet information.

#### 4.4.2 The heterogeneity of education

For individuals without the college degree, Internet use significantly increases their perception of livelihood risks, while this result is not significant at the 95% confidence level among those with a college degree. Perhaps people with a higher level of education tend to be more rational and capable to identify and judge various types of risk information on the Internet, so their perception of livelihood risks is not significantly affected by Internet information.

#### 4.4.3 The heterogeneity of income

Compared with the low-income group, the middle-income group is more likely to access the Internet resulting from the certain advantages in economic conditions, leaving them more vulnerable to the influence of Internet. And the risks faced by middle-income group not only stem from the risk events but also depend on the ability to process risk information. Due to a lack of sufficient experience and ability, they may feel lost in the complex information on the Internet with an incomplete understanding of risks. What’s worse, along with the outbreak of COVID-19 in 2020, the rapid flow of uncertain information and the increasing pressure of economic downturn have led to an amplified spread of livelihood risks in the middle-income group through the Internet. By contrast, individuals from upper-middle income group usually have a higher level of education and more stable economic foundation that endow them with better digital literacy and skills, which prevents them from excessively amplifying risk perception. As for the reason why the impact of Internet use on the risk perception of high-income group is higher than that of upper-middle group, it mainly stems from the damage caused by the epidemic to various aspects of economy. High-income individuals have a quite wide range of revenue stream and asset distribution composed of various aspects such as salary, equity, dividend, interest, bonus as well as production and operation that may be affected by various livelihood risks. In particular, these diversified sources of income inevitably suffered the impact from economic fluctuations and policy changes during the pandemic, making high-income group more sensitive to livelihood risks.

#### 4.4.4 The heterogeneity of region

The heterogeneity of region demonstrates that the impact of Internet use on the public livelihood risk perception in urban area is higher than that in rural area. Due to the differences in urban and rural network infrastructure, urban residents have more opportunities to access the Internet, bringing about higher Internet penetration, and thus the impact of Internet use on urban residents will be more prominent. This once again proves that the reason why Internet can strengthen public livelihood risk perception is that the use of Internet, that is, livelihood risk perception of those who have earlier access to the Internet is more obviously affected by the Internet use.

### 4.5 Moderation effect test

In the previous mechanism analysis, it is proposed that human capital and social capital play a moderating role in the impact of Internet use on public livelihood risk perception. On this basis, a moderating mechanism is established, and the results can be seen in Tables 7, 8.

**Table 7:** Results of the moderation effect test on human capital.

Variables	Risk_perception	Risk_perception	Risk_perception
	(1)	(2)	(3)
Internet	0.491^***^		0.650^***^
	(0.032)		(0.099)
Human_capital		−0.314^***^	−0.138
		(0.086)	(0.141)
IH			−0.270^*^
			(0.163)
Age	−0.007^***^	−0.014^***^	−0.005^***^
	(0.001)	(0.001)	(0.001)
Gender	−0.057^**^	−0.027	−0.049^**^
	(0.024)	(0.025)	(0.024)
Marriage	0.034	0.045^**^	0.036^*^
	(0.022)	(0.022)	(0.022)
Scale	0.005	−0.001	0.006
	(0.006)	(0.006)	(0.006)
Work	−0.065^**^	−0.061^*^	−0.059^*^
	(0.032)	(0.032)	(0.032)
Urban	0.048^*^	0.080^***^	0.045^*^
	(0.025)	(0.025)	(0.025)
Medical_insurance	0.052	0.054	0.050
	(0.039)	(0.040)	(0.039)
Endowment_insurance	0.051^*^	0.080^***^	0.047^*^
	(0.028)	(0.028)	(0.028)
Income	0.032^**^	0.061^***^	0.030^**^
	(0.012)	(0.012)	(0.012)
Economic status	−0.005^***^	−0.005^***^	−0.005^***^
	(0.001)	(0.001)	(0.001)
Social status	−0.067^***^	−0.075^***^	−0.063^***^
	(0.013)	(0.013)	(0.013)
Life satisfaction	−0.076^***^	−0.073^***^	−0.069^***^
	(0.015)	(0.015)	(0.015)
Confidence level about the future	0.078^***^	0.083^***^	0.085^***^
	(0.015)	(0.016)	(0.015)
Education expenditure	0.203^*^	0.243^**^	0.204^*^
	(0.110)	(0.111)	(0.110)
Social security and employment expenditure	0.394^***^	0.427^***^	0.400^***^
	(0.050)	(0.050)	(0.050)
Medical and hygienic expenditure	−0.659^***^	−0.755^***^	−0.661^***^
	(0.130)	(0.130)	(0.130)
Environmental expenditure	0.204^***^	0.225^***^	0.206^***^
	(0.042)	(0.043)	(0.042)
Housing security expenditure	−0.083	−0.089	−0.089
	(0.057)	(0.057)	(0.057)
Constant	4.180^***^	4.604^***^	4.060^***^
	(0.260)	(0.260)	(0.268)
Obs	19,084	19,084	19,084
Adj. R^2^	0.047	0.035	0.048
VIF	4.67	4.67	5.56

***, **, *indicate statistically significant at the level of 1, 5 and 10% respectively.

**Table 8:** Results of the moderation effect test on social capital.

Variables	Risk_perception	Risk_perception
	(1)	(2)
Internet		0.560^***^
		(0.063)
Social_capital	−0.065^***^	−0.053^***^
	(0.005)	(0.008)
IS		−0.020^*^
		(0.010)
Age	−0.011^***^	−0.005^***^
	(0.001)	(0.001)
Gender	−0.056^**^	−0.068^***^
	(0.024)	(0.024)
Marriage	0.049^**^	0.034
	(0.022)	(0.022)
Education	0.041^***^	0.030^***^
	(0.003)	(0.004)
Scale	0.011^*^	0.014^**^
	(0.006)	(0.006)
Work	−0.055^*^	−0.053^*^
	(0.032)	(0.032)
Urban	0.013	−0.000
	(0.025)	(0.025)
Health	−0.029^***^	−0.030^***^
	(0.011)	(0.011)
Medical_insurance	0.054	0.057
	(0.039)	(0.039)
Endowment_insurance	0.042	0.025
	(0.028)	(0.028)
Income	0.021^*^	0.004
	(0.013)	(0.013)
Economic status	−0.004^***^	−0.004^***^
	(0.001)	(0.001)
Social status	−0.050^***^	−0.041^***^
	(0.013)	(0.013)
Life satisfaction	−0.048^***^	−0.045^***^
	(0.015)	(0.015)
Confidence level about the future	0.099^***^	0.099^***^
	(0.015)	(0.015)
Education expenditure	0.213^*^	0.186^*^
	(0.110)	(0.110)
Social security and employment expenditure	0.349^***^	0.341^***^
	(0.050)	(0.050)
Medical and hygienic expenditure	−0.659^***^	−0.591^***^
	(0.129)	(0.129)
Environmental expenditure	0.196^***^	0.182^***^
	(0.042)	(0.042)
Housing security expenditure	−0.071	−0.075
	(0.057)	(0.057)
Constant	4.811^***^	4.381^***^
	(0.259)	(0.262)
Obs	19,084	19,084
Adj. R^2^	0.052	0.062
VIF	4.35	4.54

***, **, *indicate statistically significant at the level of 1, 5 and 10% respectively.

#### 4.5.1 The moderation effect of human capital

In the Section 4.2, the control variables in benchmark regression include education level and health status overlapped with the components of human capital, which will trigger a serious multicollinearity problem if continuing to include them when testing the moderation effect of human capital. Specifically, when the education level and health status serve as control variables, the variance inflation factor (VIF) of the model soars to 20.74, indicating significant multicollinearity. To address this issue, we eliminate these two variables from the model and find the VIF down to 4.67, which means that the multicollinearity problem in the model has been effectively alleviated. This adjustment avoids parameter estimation deviation caused by multicollinearity, and also conforms to the mutual independence of control variables, ensuring the robustness and reliability of model estimation.

According to the results in Table 7: firstly, after inclusion of the interaction term between the Internet and human capital (IH), there still remains the amplification effect of Internet on public livelihood risk perception, which is consistent with the previous conclusion; secondly, the coefficient of IH is significantly negative, which suggests that the amplification effect is alleviated with the improvement of human capital. Thus, H3 is supported.

#### 4.5.2 The moderation effect of social capital

Similarly, the results in Table 8 show that the coefficient of the interaction term between the Internet and social capital (IS) is significantly negative, indicating that social capital has a significant negative regulation in the process when the Internet amplifies public livelihood risk perception. Online social networks are be squeezed out by the rich offline interpersonal interaction, community participation, and other public activities, which reduces individual dependence on the Internet, then alleviating the amplification of public livelihood risk perception caused by Internet use. H4 of this study is confirmed.

### 4.6 Robustness

#### 4.6.1 Long-term robustness test

After further examination of the data from 2014 to 2018 with the same control variables as before, we have revealed the dynamic changes in the amplification effect of Internet on public livelihood risk perception and come to the following conclusion: the amplification effect of Internet on public livelihood risk perception has existed for a long time with a steady downward trend (see Table 9), which indicates that the public has become more rational to evaluate and absorb Internet information over time, so that the negative impact of risk amplification can be minimized. However, we notice that the amplification effect may be intensified in the crisis period. The outbreak of COVID-19 in 2020 brought an unprecedented impact to society with the random spread of various risk information through the Internet sparking the infodemic, globally inducing psychological anxiety as well as social panic, and temporarily intensifying the public livelihood risk perception. Nevertheless, from a long-term perspective, this effect has not caused risk perception to deviate significantly from an appropriate range. This reflects the adaptability and resilience of society to the impact of information on the Internet in the face of public crisis.

**Table 9:** Baseline regression results from 2014 to 2018.

Year	2014	2016	2018
Variables	Risk_perception	Risk_perception	Risk_perception
	(1)	(2)	(3)
Internet	0.984^***^	0.746^***^	0.420^***^
	(0.025)	(0.014)	(0.026)
Controls	Yes	Yes	Yes
Constant	2.007^***^	5.311^***^	4.853^***^
	(0.216)	(0.129)	(0.234)
Obs	26,868	27,764	20,606
Adj. R^2^	0.120	0.211	0.075

Meanwhile, this also denotes that the amplification effect may eventually tends toward a state of stability in which the Internet is no longer just an amplifier of risk perception, but a positive alarm to society that improves the public awareness of risk prevention, then providing support for the sustainable development and social stability. The above findings validate H2. Similarly, the moderation effect of human capital and social capital also has long-term existence during this process (see Tables 10, 11), which proves H5.

**Table 10:** Results of the moderation effect test on human capital from 2014 to 2018.

Year	2014			2016			2018		
Variables	Risk_perception	Risk_perception	Risk_perception	Risk_perception	Risk_perception	Risk_perception	Risk_perception	Risk_perception	Risk_perception
	(1)	(2)	(3)	(4)	(5)	(6)	(7)	(8)	(9)
Internet	0.994^***^		1.481^***^	0.796^***^		1.256^***^	0.437^***^		0.561^***^
	(0.025)		(0.066)	(0.014)		(0.026)	(0.026)		(0.042)
Human_capital		−0.890^***^	−0.074		−4.814^***^	−0.948^***^		−0.087^*^	−0.070
		(0.145)	(0.152)		(0.201)	(0.203)		(0.049)	(0.067)
IH			−1.754^***^			−1.509^***^			−0.334^***^
			(0.219)			(0.067)			(0.091)
Controls	Yes	Yes	Yes	Yes	Yes	Yes	Yes	Yes	Yes
Constant	2.052^***^	2.180^***^	1.885^***^	5.391^***^	4.181^***^	4.898^***^	4.998^***^	5.263^***^	4.974^***^
	(0.212)	(0.219)	(0.213)	(0.128)	(0.138)	(0.131)	(0.229)	(0.231)	(0.230)
Obs	26,868	26,868	26,868	27,764	27,764	27,764	20,606	20,606	20,606
Adj. R^2^	0.119	0.069	0.122	0.206	0.126	0.224	0.074	0.061	0.075

**Table 11:** Results of the moderation effect test on social capital from 2014 to 2018.

Year	2014		2016		2018	
Variables	Risk_perception	Risk_perception	Risk_perception	Risk_perception	Risk_perception	Risk_perception
	(1)	(2)	(3)	(4)	(5)	(6)
Internet		1.014^***^		0.768^***^		0.558^***^
		(0.044)		(0.025)		(0.045)
Social_capital	−0.079^***^	−0.061^***^	−0.045^***^	−0.031^***^	−0.058^***^	−0.040^***^
	(0.004)	(0.004)	(0.002)	(0.003)	(0.004)	(0.006)
IS		−0.016^*^		−0.009^**^		−0.031^***^
		(0.008)		(0.004)		(0.008)
Controls	Yes	Yes	Yes	Yes	Yes	Yes
Constant	2.486^***^	2.230^***^	5.189^***^	5.378^***^	5.208^***^	4.898^***^
	(0.221)	(0.216)	(0.134)	(0.129)	(0.234)	(0.233)
Obs	26,868	26,868	27,764	27,764	20,606	20,606
Adj. R^2^	0.084	0.130	0.145	0.218	0.073	0.085

#### 4.6.2 Alternative risk perception measurement robustness test

We use principal component analysis to form a new comprehensive indicator of livelihood risk perception recorded as P_risk_perception. Table 12 presents the regression results of Internet use on the P_risk_perception. The results demonstrate that, similar to Risk_perception, Internet use has a significant and long-term facilitation effect on P_risk_perception, which also tends to decline, but recovers somewhat when the public health emergency occurs. This finding once again confirms the long-term robustness of Internet in enlarging the livelihood risk perception.

**Table 12:** Estimation results of P_risk_perception.

Year	2014	2016	2018	2020
Variables	P_ risk_perception	P_ risk_perception	P_ risk_perception	P_ risk_perception
Internet	0.729^***^	0.474^***^	0.279^***^	0.282^***^
	(0.014)	(0.012)	(0.017)	(0.020)
Controls	Yes	Yes	Yes	Yes
Adj. R^2^	0.119	0.210	0.075	0.049

## 5 Conclusions and policy implications

This study is based on four rounds of CFPS data from 2014 to 2020 to examine the impact of Internet use on public livelihood risk perception. The main research conclusions are as follows: First, the amplification effect generated by the spread of risk information through the Internet can raise the individual awareness of livelihood risks, thus forming a strengthening effect of Internet use on livelihood risk perception. This conclusion remains robust after using the instrumental variables and difference-in-differences estimation to address endogeneity as well as different methods to construct comprehensive indicators of public livelihood risk perception. Second, there exit the marked disparities in the amplification effect of Internet on livelihood risk perception among different groups. Specifically, this amplification effect exhibits more obvious in the middle and elder age, individuals without university degree, middle-income group as well as urban residents. More than that, it is discovered that human capital and social capital have a negative moderation effect on public livelihood risk perception. These impact mechanisms show consistency across different rounds of surveys, demonstrating the robustness of long-term mechanisms. Last but not least, this study reveals the dynamic relationship between information dissemination and individual perception, that is, the amplification effect of Internet on livelihood risk perception is steadily declining, but the burst of public crisis events may cause a temporary recovery.

The amplification effect of the network on social risks is an objective reality. There is need to prevent the Internet and its related information technology from constantly widening the social gap as well as aggravating the phenomenon of digital inequality, especially for people with low education who may be still in the state of deficient information. However, it should be pointed out that this kind of amplification dissemination can also have certain positive effects: (1) It satisfies the public’s right to learn the truth and avoids greater social panic caused by lack of information transparency. (2) It mobilizes the public participation, and especially in the face of public crisis, the long tail effect of Internet gathers the participation of all nodes to provide strong support for the resolution of incidents. At the same time, we should also recognize that appropriate risk perception can promote individual effort and social progress. Therefore, we need to deal with the social risk amplification of the network with rationality. Relevant management departments should adopt the reasonable measures to maximize the positive effect and minimize the negative effect as much as possible.

Firstly, the digital skills of individuals should be further improved, especially those low-skilled with low education as well as low income deserve focused attention. In order to make them become new pattern laborers that meet the profession demands of the digital age, various measures including the training of Internet skills and professional retraining should be taken to break through the difficulties produced by a composition of intelligent divide on digital divide, and reduce public sense of crisis from the squeeze of employment opportunities caused by the development of digital technology. Additionally, the characteristic of Internet to break the time and space limitations contributes to the flexible employment, and thereby it is crucial to take full advantage of this opportunity to widen the way of raising income so as to protect the livelihood of people and decrease social inequality with the improvement of living quality and ability to resist risks in low-income group.

Secondly, individuals should improve the level of human capital through the Internet platform, especially in the age of excessive information overload on the Internet, in order to avoid the disadvantages in the dissemination of network information, digital literacy should be attached importance in cultivation of the public’s ability to identify and judge risk information on the Internet. In addition, to limit the recommendation weight of illegal incitements, we can also learn from the Digital Services Law of the European Union, allowing Internet users to turn off personalized recommendation as well as cultivating social consensus in the long term.

Thirdly, the transmission of policy information should be optimized through the Internet platform. For example, establishing online public consultation during the planning and practice process of policy can promote the active engagement and feedback, improve the transparency of government work with public acceptance, and conduce to the adjustment and optimization of policy in a timely manner. In the meantime, statistical data and policy related to livelihood can update to the public because of the broad reach and immediacy of the Internet, along with the visual data easy to comprehend, can assist the public in better understanding the relevant information, ensuring the effectiveness and accessibility of information. Especially when emergencies (such as public health events and natural disasters) occur, the government needs to actively release authoritative information through the Internet platform, but it needs to avoid panic caused by information overload at the same time. We can learn from the risk communication principles of WHO, which include ensuring trust and transparency, early announcement and planning, and listening to public voices and involving them. In addition, social media platforms can adjust recommendation algorithms during the crisis, reduce the recommendation of illegal incitements, and put forward some services such as psychological assistance.

Finally, the balanced development of traditional media and emerging media should be supported to provide a variety of channels for obtaining information. For instance, social organization should play the role as a bridge, establish the information center at the community level to supply various public resources in the field of livelihood and create more opportunities for information acquisition to reduce over-reliance on the Internet.

The amplification effect of Internet on public livelihood risk perception is a global phenomenon, but the governance strategies of different countries vary depending on the political system, cultural traditions, and digital development level. Those are policy implications proposed from two dimensions: common patterns and individual differences, based on this study and international experience. While maintaining the dividend of Internet development and governance effectiveness, China can absorb international experience, build an accurate, inclusive and universal risk governance system, and provide a “China solution” for global digital governance by identifying commonness and individuality.

## 6 Limitations

There are certain limitations in this study: the measurement of the independent variable “Internet use” employs a simplified binary variable (whether to use the Internet or not), rather than a more accurate indicator of use frequency or intensity. Although this measurement method can verify the basic effect of Internet access, it is difficult to capture the difference in risk perception caused by heterogeneity of use behavior, which conceals the potential nonlinear relationship between the intensity of Internet use and risk perception. According to these limitations, future research can be expanded from the following directions: Firstly, it is recommended to use continuous variables or multidimensional indicators to measure Internet use behavior, including frequency, duration and purpose, to more accurately assess the differences of amplification effect among groups with different intensity of Internet use. Secondly, it is worth exploring the impact mechanism of different Internet use purposes on risk perception. Finally, it is possible to consider establishing a comprehensive digital engagement index that integrates multiple dimensions such as access, skills, and usage intensity to gain a more comprehensive understanding of the formation mechanism of risk perception in the digital age. These improvements will help deepen our understanding of the relationship between the Internet and risk society, and provide the scientific evidence for formulating more precise digital governance policies.

## Data Availability

Publicly available datasets were analyzed in this study. This data can be found here: http://www.isss.pku.edu.cn/cfps.

## Footnotes

^1^Beck created the theory of risk society and pointed out that the perception and management of risks in modern society are central issues.

^2^According to the 47th Statistical Report on the Development of Internet in China published by CNNIC, the Internet penetration has reached 70.4% by the end of 2020.
